# Fibrogenic Secretome of Sirtuin 1-Deficient Endothelial Cells: Wnt, Notch and Glycocalyx Rheostat

**DOI:** 10.3389/fphys.2018.01325

**Published:** 2018-09-21

**Authors:** Mark Lipphardt, Hassan Dihazi, Gerhard A. Müller, Michael S. Goligorsky

**Affiliations:** ^1^Departments of Medicine, Physiology and Pharmacology, New York Medical College, Valhalla, NY, United States; ^2^Clinic for Nephrology and Rheumatology, Göttingen University Medical Faculty, Georg-August-Universität Göttingen, Göttingen, Germany

**Keywords:** endothelial secretome, DKK3, Jagged-1, syndecan-4, fibrosis

## Abstract

Sirtuins (SIRT) are ubiquitous histone and protein deacetylases and a member of this family, SIRT1, is the best-studied one. Its functions in endothelial cells encompass branching angiogenesis, activation of endothelial nitric oxide synthase, regulation of proapoptotic and proinflammatory pathways, among others. Defective SIRT1 activity has been described in various cardiovascular, renal diseases and in aging-associated conditions. Therefore, understanding of SIRT1-deficient, endothelial dysfunctional phenotype has much to offer clinically. Here, we summarize recent studies by several investigative teams of the characteristics of models of global endothelial SIRT1 deficiency, the causes of facilitative development of fibrosis in these conditions, dissect the protein composition of the aberrant secretome of SIRT1-deficient endothelial cells and present several components of this aberrant secretome that are involved in fibrogenesis via activation of fibroblasts to myofibroblasts. These include ligands of Wnt and Notch pathways, as well as proteolytic fragments of glycocalyx core protein, syndecan-4. The latter finding is crucial for understanding the degradation of glycocalyx that accompanies SIRT1 deficiency. This spectrum of abnormalities associated with SIRT1 deficiency in endothelial cells is essential for understanding the origins and features of endothelial dysfunction in a host of cardiovascular and renal diseases.

## Introduction

The broad spectrum of deacetylation targets and dense network of metabolic connections of SIRT1 (Haigis and Guarente, 2006) have contributed to the steadfast interest to the consequences of its loss or gain of function. Endothelial SIRT1 deficiency is a common companion of various cardiovascular, metabolic and renal diseases, as well as of aging (Haigis and Guarente, 2006; Potente et al., 2007; Chen et al., 2012; Maizel et al., 2014; Vasko et al., 2014). All endothelial functions (deterrent of leukocytes, regulation of permeability and vasomotion, angiogenesis, platelet adhesion and coagulation) become perturbed in SIRT1 deficiency, thus being responsible for global endothelial dysfunction. Below we shall briefly catalog some sequelae of endothelial SIRT1 deficiency and then focus on the aberrant profibrogenic secretome of dysfunctional endothelial cells.

The sirtuin (SIRT) family consists of seven mammalian NAD-dependent histone deacetylases. SIRT 1 and 2 are expressed in the nucleus and the cytosol, SIRT 3–5 have been identified as mitochondrial proteins and SIRT 6 and 7 are solely expressed in the nucleus (Haigis and Guarente, 2006). It has been demonstrated that among those family members SIRT1 plays a critical role in the control of cellular differentiation and senescence, as well as balancing metabolic pathways, in a broad variety of tissues (Blander and Guarente, 2004). There is a remarkably high expression level of SIRT1 in endothelial cells where it regulates a host of functions, such as activity of nitric oxide synthase, cell senescence, and autophagy (Borradaile and Pickering, 2009). In a pioneering study by Potente et al. (2007) the phenotype of mammalian and zebrafish endothelial cells expressing SIRT1 with disabled deacetylase domain was investigated to set a precedent for use of this model system in future studies. Those studies represent a working model for the current review.

## SIRT1-Deficient Endothelial Cells – Phenotype

SIRT1-null mice exhibit high rates of perinatal mortality and those that survive show developmental deficits, such as growth retardation, sterility, impaired DNA damage response, genome instability, impaired lipid metabolism and liver steatosis, defective immune responses, impaired cognitive functions, and stem cell differentiation, among other abnormalities, which collectively hamper their use (Wang et al., 2008; Boily et al., 2010; Xu et al., 2010; Ou et al., 2011). Therefore, the ways to overcome this problem include either the use of conditional knockout, or heterozygote mice, or, more often, cell-specific mutagenesis of SIRT1. Certainly, a degree of artificiality could be introduced by the cell-specific knockout, which presumably leaves all other cells intact. As we shall see from the following discussion, this is not the case: endothelial deletion of SIRT1 deacetylase-encoding domain profoundly affects neighboring cells and tissues.

One of the dire characteristics of SIRT1-deficient endothelial cells is the reduced endothelium-dependent vasorelaxation. Functional SIRT1 deacetylates the endothelial nitric oxide synthase (eNOS) on lysines 496 and 506 in the calmodulin-binding domain of eNOS. With the deacetylation of eNOS its activity is increased leading to elevated levels of bioavailable endothelial nitric oxide (Mattagajasingh et al., 2007). Endothelial SIRT1-deficiency is also characterized by microvascular rarefaction, which can already be seen at a basal state without any endogenous or exogenous insult. The level of microvascular rarefaction increases significantly after damage (Kida et al., 2016). Potente et al. (2007) have shown that endothelial SIRT1-deficiency impedes sprouting angiogenesis and branching morphogenesis with simultaneous down-regulation of genes involved in blood vessel growth and vascular remodeling. Among transcriptomic signatures of endothelial cells with the silenced SIRT1 these authors have discovered phosphoinositide-3-kinase, Fms-related tyrosine kinase (Flt1), bone morphogenic protein 4, platelet-derived growth factor beta, TGF-beta-activated kinase 1, matrix metalloproteinase 14, ephrin receptor EphB2 (all downregulated), sonic hedgehog homolog (Shh), angiopoietin-2, a disintegrin and metalloproteinase domain 17 (ADAM17) (all upregulated), among others. They further demonstrated that SIRT1 deacetylates the forkhead transcription factor Foxo1, a central negative regulator of blood vessel growth to inhibit its anti-angiogenic activity (Potente et al., 2007). Impaired angiogenic sprouting and matrilytic activity has also been noted by Vasko et al. (2014) by culturing aortic rings from mice pretreated with a SIRT1-inhibitor. Echocardiographic studies by Maizel et al. (2014) revealed development of diastolic dysfunction on the background of myocardial vascular rarefaction in mice lacking endothelial SIRT1 deacetylase function. Another characteristic of SIRT1-deficient endothelial cells is their vulnerability to stress-induced premature vascular senescence (SIPS). As dysfunctional endothelial cells exhibit increased lysosomal permeability and leakage of cathepsins, Chen et al. (2012) demonstrated a cathepsin-induced proteolytic cleavage of SIRT1 leading to its depletion, and consequently to cell stress and SIPS.

## SIRT1 Deficiency Predisposing to Renal Fibrosis

The described characteristics and spectrum of functions explain the association of endothelial SIRT1 deficiency with impaired vasoreactivity and increased numbers of prematurely senescent endothelial cells. However, mice with endothelial SIRT1-deficiency have also been shown to have a higher susceptibility to develop fibrosis. Vasko et al. (2014) have used genetically engineered mice with a deletion of the exon 4 (encoding the deacetylase domain) in endothelial SIRT1 (hereafter abbreviated as SIRT1^endo^-/-) and have demonstrated that those mice not only develop vasculopathy and premature senescence, but also tubulointerstitial fibrosis at an early age. One of the mechanistic links presented is the observation that endothelial SIRT1-deficient mice have lower expression of the master matrix metalloproteinase, MMP-14. Lower MMP-14 leads to impaired degradation of deposited matrix proteins, and as a result of reduced matrilytic activity, endothelial cells lose their ability to navigate within the interstitial matrix and recover metabolism and regeneration of the affected organs (Oh et al., 2001; Riggins et al., 2010; Vasko et al., 2014). Pharmacologic stimulation of MMP-14 expression, using a SIRT1-independent strategy, is able to lower tubulointerstitial fibrosis in SIRT1^endo^-/- mice, thus confirming the role of MMP-14 in the development of tubulointerstitial fibrosis in those mice (Vasko et al., 2014). Of note, a similar downregulation of MMP-14 in SIRT1 deficiency has been noted by Potente et al. (2007).

Another mechanistic link on how SIRT1 deficiency leads to renal fibrosis has been proposed by Huang et al. Depletion of SIRT1 increases the acetylation level of Smad3 and consequently increases its transcriptional activity following activation by TGF-β1. On the other hand, TGF-β1-induced extracellular matrix expression was attenuated by elevated levels of SIRT1, supporting the idea of protective role of SIRT1 in the development of renal fibrosis due to its action on TGF-β/Smad3 signaling (Huang et al., 2014).

## The Secretome of SIRT1-Deficient Renal Microvascular Endothelial Cells

Genetic manipulation of relevant gene expression in endothelial cells has brought about intriguing findings. On the one hand, studies performed by Xavier et al. demonstrated that mice with endothelium-specific heterozygous TGFβ receptor II knockout (TGFβRII^endo^+/-) are protected against tubulointerstitial fibrosis (induced in two different models, unilateral ureteral obstruction (UUO) and chronic phase of folic acid nephropathy), have a better preservation of renal microvasculature, show a better renal blood flow, and have a less profound tissue hypoxia than TGFβRII^endo^+/+ control mice. TGFβRII^endo^+/- mice also show suppressed endothelial-to-mesenchymal transition in these fibrogenic models (Xavier et al., 2015). On the other hand, as mentioned above, SIRT1^endo^-/- mice develop exaggerated fibrosis under basal conditions and following UUO and folic acid nephropathy (Vasko et al., 2014). The finding that SIRT1^endo^-/- mice are more susceptible to development of tubulointerstitial fibrosis, in contrast to TGFβRII^endo^+/- mice that are protected against it, supports the concept that distinct messages from the endothelium are instructive for fibroblasts. It also prompted us to compare endothelial secretomes, as the only distantly acting variable with the potential to explain the distinct end-results. A fraction of proteins, which are secreted to the extracellular compartment is defined as the cell secretome. The secretome is very specific and depends on the types of cells and tissues and the secretomic response differs based on the physiologic states and pathologic conditions.

The progression of tissue fibrosis going side by side with microvascular rarefaction still does not have a molecular explanation. One possible way to explain multiple confirmatory observations is by invoking the communication between endothelial cells and fibroblasts via secretory products. In order to analyze proteomic signatures of secretory products of renal microvascular endothelial cells (RMVEC), we obtained and analyzed conditioned media of high-purity populations of RMVEC from control wild-type, SIRT1^endo^-/- and TGFβRII^endo^+/- mice using unbiased, non-targeted MS/MS, after they were exposed to a vehicle or TGFβ for 48 hours (Lipphardt et al., 2017). 332 non-redundant proteins, which belong to diverse categories, were detected. Out of the 332 proteins, those secreted only by SIRT1^endo^-/- vis-a-vis TGFβRII^endo^+/- are of special interest, since they could theoretically be responsible for the pro- and anti-fibrogenic phenotype of the respective mice. Some of these findings are presented in **Table 1**. Detailed list of identified protein in the endothelial secretome can be found in **Supplementary Table 1**.

**Table 1:** Details of three selected proteins of the secretome of SIRT1–/– RMVEC.

Protein name	Gene name	Accession number	Molecular weight (kDa)	Sequence coverage (%)	Unique peptides
Dickkopf-related protein 3	DKK3	Q9QUN9	38.4	24	8
Jagged-1	Jag1	Q9QXX0	134.2	17	17
Syndecan-4	Sdc4	O35988	21.5	15	4

*A partial list of proteins of interest identified as secreted at a high level in SIRT1-/- RMVEC treated with TGFß-1 compared to TGFR2+/- RMVEC subjected to the similar treatment. Gene name, calculated molecular weight and accession number are given. Additional MS/MS information, e.g., the number of unique peptides that were sequenced through MS/MS and the achieved sequence coverage are provided*.

## Agonists of Notch and Wnt Pathways in the Secretome

**FIGURE 1:**
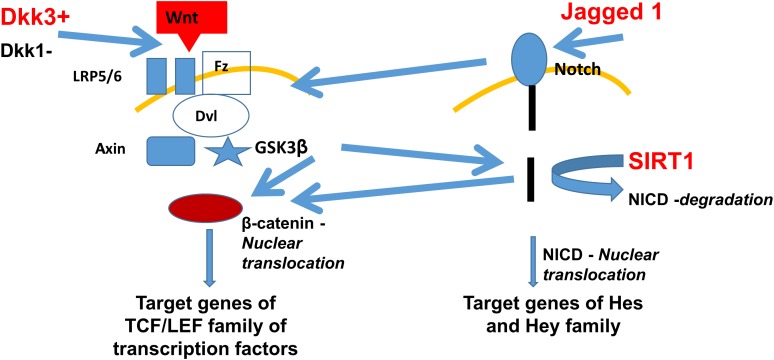
Fibrogenic secretome pathways implicated in the development of fibrosis and their dependence on SIRT1. Secretomic findings of DKK3 and Jagged1 are shown in red. DKK3 induces the Wnt pathway activating the frizzled receptor with its co-receptors LRP5/6. Further activation of the GSK3β leads to β-catenin nuclear translocation and therefore inducing the TCF/LEF family, target genes of the Wnt pathway. Jagged1 activates the Notch pathway with further NICD nuclear translocation and activation of Hes and Hey genes. Lack of SIRT1 leads to less degradation of NICD, which also leads to more β-catenin nuclear translocation, combining the Wnt and Notch pathway. Abbreviations: Wnt, wingless-related integration site – a signaling glycoprotein; Fz, frizzled receptor – a family of G protein-coupled receptor proteins serving as Wnt receptors; Disheveled – a family of cytoplasmic phosphoproteins downstream of frizzled receptors, which are involved in canonical and non-canonical Wnt signaling; NICD, notch intracellular domain; GSK3β, glycogen synthase kinase 3 beta; TCF/LEF family is a group of transcription factors involved in the Wnt signaling pathway; Hes, hairy and enhancer of split transcription factor protein; and Hey hairy/enhancer-of-split related transcription factor protein.

Intriguingly, Jagged1 (a ligand of the Notch pathway) and Dickkopf3 (DKK3) (a putative ligand of the Wnt pathway) were found among the proteins exclusively secreted by SIRT1^endo^-/- mice upon stimulation with TGFβ. Both pathways previously have been implicated in the development of fibrosis, are mechanistically interconnected (see **Figure 1**), Notch signaling is SIRT1-dependent [it deacetylates Notch intracellular domain, NICD, and targets it to proteasomal degradation (Guarani et al., 2011)] and therefore warranted our further investigation. Besides the important roles in carcinogenesis, heart function and bone metabolism, among others, Wnt signaling and the Dickkopf family proteins have a vast impact during the embryogenesis of the kidney and exert a key role in the development or progression of chronic kidney disease (CKD) and kidney fibrosis. It has been shown that Wnt proteins can act on unstimulated mesenchymal cells and program them to differentiate into epithelial cells (Boyle et al., 2011). Indeed, Wnt-responsive cells have been found in the renal medulla and renal tubules of adult mice and have most likely the capability to act as progenitor or stem cells (Oliver et al., 2004; Rinkevich et al., 2014). The fact that many Wnt proteins are upregulated after application of acute ischaemia-reperfusion injury, is consistent with their ability to promote cell regeneration (Lin et al., 2010; Kawakami et al., 2013).

Regarding the Wnt pathway in the development of kidney fibrosis, 16 out of 19 Wnt proteins and 8 out of 10 Frizzled receptors have been found to be elevated in renal tubular cells in kidney fibrosis due to UUO (He et al., 2009). Zhou et al. (2012, 2013) showed that genetic overexpression of active β-catenin in tubular cells induces kidney fibrosis and epithelial-mesenchymal transition (EMT).

Among the DKK protein family, the role of DKK3 and its interactions with the Wnt pathway is controversial. Dickkopf1 (DKK1) is a well-studied Wnt antagonist and He et al. (2009) demonstrated that the injection of a vector encoding for DKK1 reduced tubulointerstitial fibrosis and β-catenin accumulation. DKK3, on the other hand, has been shown to act as a pro-survival signal by positively modulating the Wnt pathway (Nakamura and Hackam, 2010). DKK3 also has been described as a cytokine capable of inducing stem cell differentiation (Wang et al., 2015), as a tumor suppressor (Veeck and Dahl, 2012) and as an inhibitor of VEGFR2/Akt/mTOR signaling (Kim et al., 2015). Recently, DKK3 was found to be a tubular epithelia-derived mediator of kidney fibrosis with decreased tubular atrophy and interstitial matrix accumulation after blockade of DKK3 in different mouse models of kidney fibrosis (Federico et al., 2016). Similarly, proximal tubules with tamoxifen-induced Wnt1 expression have profound interstitial myofibroblast activation and proliferation and increased extracellular matrix proteins production (Maarouf et al., 2016). We showed that DKK3 is able to convert fibroblasts into myofibroblasts with DKK3 being secreted by SIRT1 deficient endothelial cells (Lipphardt et al., 2018a). We also investigated the relations between DKK1 and DKK3 and learned that DKK3 is only able to activate fibroblasts to acquire myofibroblastic phenotype at low levels of DKK1. With escalating levels of ambient DKK1 the conversion of fibroblasts into myofibroblasts is averted. Those observations would argue that cells closest to dysfunctional endothelial cells, for example pericytes, are most impacted by DKK3 secreted from dysfunctional endothelium. The data also indicate the existence of rheostat-like relations between DKK1 and DKK3. Our endothelial findings in conjunction with the existing data on Wnt and DKK3 secretion from stressed renal tubular epithelial cells (Federico et al., 2016; Maarouf et al., 2016) lead us to assume that the renal microvascular endothelium serves as a fine-tuned modulator of Wnt agonists and antagonists reaching renal fibroblasts.

The Notch pathway has been established to be of significant importance during kidney development (Sirin and Susztak, 2012). For instance, human kidney developmental abnormalities have been observed due to mutations of Jagged1, a Notch ligand, and Notch2, a Notch receptor (Penton et al., 2012). Upon completion of developmental programs, the resident stem cell population harbors the most active Notch signaling among adult organs.

Regarding the impact of Notch signaling in the development of kidney fibrosis it is crucial to mention that the transcript and protein levels of members of the Notch pathway are highly elevated in patients with CKD, such as diabetic nephropathy, lupus nephritis and focal segmental glomerulosclerosis. It has been demonstrated also that Notch1 expression in renal tubules is associated with tubulointerstitial fibrosis and decreased kidney function in patients with CKD (Murea et al., 2010). Supporting the findings of the above mentioned study, Bielesz et al. described increased epithelial cell proliferation and tubular epithelial dedifferentiation after induced cell-specific expression of cleaved Notch1 in tubular epithelial cells, concluding that the expression of Notch receptors in tubular epithelial cells is capable of inducing EMT. They further investigated the genetic deletion of Rbpj, the central transcriptional mediator of Notch signaling in proximal tubular epithelial cells, and found reduced tubulointerstitial fibrosis following folic acid nephropathy (Bielesz et al., 2010). A different approach in deleting Notch3 showed protection against tubulointerstitial fibrosis with decreased numbers of αSMA positive cells following UUO (Djudjaj et al., 2012).

Among the ligands for the Notch pathway we found Jagged1, which was exclusively secreted by SIRT1-deficient endothelial cells after TGFβ stimulation. 2-D culture studies also showed higher endothelial-mesenchymal transition after exposure to Jagged1 or Jagged2, especially in combination with TGFβ1 (unpublished data). Additional experiments confirmed the activation of the Notch pathway by demonstrating NICD (intracellular domain of the Notch protein) and αSMA-positive RMVEC (unpublished data). In order to investigate the combined effects of Jagged1 on renal fibroblasts and RMVEC we used a co-culture system utilizing microfluidic devices based on previously published protocols (Kim et al., 2013). Once the RMVEC and the renal fibroblasts were exposed to Jagged1 we observed a decrease in total vessel length, reduced number of bifurcations along with reduced percentage area occupied by capillary-like structures (unpublished data). We also detected a greater extent of endothelial-mesenchymal transition and fibroblast-to-myofibroblast conversion, as judged by the appearance of α-SMA-GFP fluorescence. Those results are in accord with our previous study showing that endothelial SIRT1-deficiency leads to enhanced microvascular rarefaction and fibrosis through activation of the Notch pathway (Kida et al., 2016). These findings, however, should be interpreted with caution, as Notch activating ligands are usually presented in immobilized form of *cis*- or *trans*-position. Kopan and Ilagan (2009) specifically discuss potential contribution of diffusible ligands and propose that they could be immobilized by extracellular matrix proteins “to provide sufficient leverage to unfold/dissociate the negative regulatory region and activate Notch.”

**FIGURE 2:**
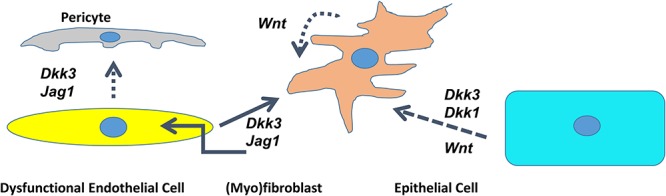
Cross talk between dysfunctional endothelial cells, pericytes, epithelial cells, and (myo)fibroblasts through DKK1/DKK3/Wnt/Jagged1 in the kidney. Schematic presentation of the interaction of different cell lines mediated by agonists of the Wnt- and Notch pathway. Dotted line – deduced conclusion. Dashed line – based on the published literature (Federico et al., 2016; Maarouf et al., 2016).

In conclusion, it appears that microenvironmental signals deriving from dysfunctional endothelial cells mediate the fate of (myo)fibroblasts. Endothelial cells deficient in SIRT1 secrete DKK3 and Jagged1 and induce the conversion of fibroblasts into myofibroblasts and contribute to the dysfunctional endothelium-dependent facilitation of tissue fibrosis, which we termed, the “third pathway” of fibrogenesis (**Figure 2**) (Lipphardt et al., 2017).

## Autocrine Effects of Endothelial Secretome

Having demonstrated the effects of DKK3 and Jagged1 secreted from dysfunctional endothelial cells exerting paracrine effects on renal fibroblasts, it was only plausible to expect their autocrine effects. In experiments on DKK3 application to cultured RMVEC we found that DKK3 significantly increased the amount of RMVEC undergoing endothelial-mesenchymal transition (Lipphardt et al., 2018a). We also showed that RMVEC cultured on matrigel exhibited reduced total vessel length, percentage area occupied by capillary-like structures, and limited number of bifurcations after treatment with DKK3 (Lipphardt et al., 2018a). As mentioned earlier, Jagged1 is also capable of inducing endothelial-mesenchymal transition and impairing angiogenesis. Dysfunctional endothelial cells not only contribute to fibrogenesis by sending microenvironmental signals to neighboring cells but also by self-reprogramming leading to enhanced endothelial-mesenchymal transition.

## Endothelial Secretome and Glycocalyx

The endothelial glycocalyx consists of hyaluronic acid cords, heparan sulfate chains and a mixture of dermatan, keratan, and chondroitin sulfates, which give the endothelial glycocalyx a net negative charge (Reitsma et al., 2007). The scaffold of the endothelial glycocalyx contains two families of proteoglycans: syndecans 1-4 and glypicans 1-6. The endothelial glycocalyx mediates the interaction between extracellular molecules, circulating cells and endothelial cells, regulates mechanosensing and vasorelaxation, coagulation, and serves as a passive barrier to water and solutes. The endothelial glycocalyx has a high vulnerability and is degraded in the presence of various stressors, such as endotoxins, ischemia/hypoxia/reperfusion, or oxidative stress. We observed that endothelial SIRT1-deficiency causes a decline in the endothelial glycocalyx already at a basal state, which is due to a higher level of shedding of syndecan-4, the main proteoglycan of the endothelial glycocalyx (Lipphardt et al., 2018b). We found this protein to be exclusively secreted by SIRT1-deficient endothelial cells upon stimulation with TGFβ was syndecan-4. We established the link between enhanced NF-κB signaling in SIRT1-deficient endothelial cells, which consequently leads to an elevation in syndecan-4 synthesis. This increase in syndecan-4 synthesis is driven by the presence of a NF-κB response element in the promoter region of Synd4 (Strand et al., 2013; Lipphardt et al., 2018b). In addition to the enhanced NF-κB signaling in SIRT1-deficient endothelial cells and therefore elevated syndecan-4 synthesis, we also showed an increased oxidative stress in SIRT1-deficient RMVEC. Increased oxidative stress may explain the activation of the major sheddase ADAM17 (its activity is regulated by 2 vicinal cysteine clusters), which leads to an enhanced shedding of syndecan-4, consequently leading to the decline of the endothelial glycocalyx (Wang et al., 2009; Lipphardt et al., 2018b). Remarkably, the shed ectodomains of Synd4 exert pro-fibrogenic effect, as we have shown by injecting ectodomain protein subcapsularly to the intact kidneys and detecting the appearance of picrosirius red staining (Lipphardt et al., 2018b). Hence, the excessive degradation of the endothelial glycocalyx can be viewed as another effect of the secretome of dysfunctional endothelial cells and may explain in part the phenotype of SIRT1-deficient endothelial cells discussed earlier.

## Conclusion

The rich metabolic network of SIRT1 is at the core of pathologic manifestations of its deficiency in endothelial cells. It not only disrupts the homeostatic processes in these cells, but also affects their functions leading to development of endothelial dysfunction. In addition, endothelial SIRT1 deficiency distorts the normal angiocrine profile of secreted molecules maintaining the structural integrity of organs, thus acquiring secretomic components, which are capable of activating fibroblasts and promoting tissue fibrosis. Furthermore, studies of the secretome of SIRT1-deficient cells revealed that a key component of endothelial glycocalyx is shed, its fragment is pro-fibrogenic, whereas the aberrant composition of endothelial glycocalyx could further exacerbate endothelial dysfunction. These findings provide added support to the therapeutic interventions to correct SIRT1 expression and activity.

## Author Contributions

ML designed the studies, performed the experiments, analyzed the data, and wrote and approved the manuscript. HD designed the studies, performed the experiments, analyzed the data, and approved the manuscript. GM designed the studies and approved the manuscript. MG designed the studies and wrote and approved the manuscript.

## Conflict of Interest Statement

The authors declare that the research was conducted in the absence of any commercial or financial relationships that could be construed as a potential conflict of interest.

## Abbreviations

ADAM17: a disintegrin and metalloproteinase domain 17
CKD: chronic kidney disease
DKK1: Dickkopf1
DKK3: Dickkopf3
EMT: epithelial-mesenchymal transition
eNOS: endothelial nitric oxide synthase
Flt1: Fms-related tyrosine kinase
NICD: Notch intracellular domain
RMVEC: renal microvascular endothelial cells
Shh: sonic hedgehog homolog
SIPS: stress-induced premature vascular senescence
SIRT1: Sirtuin1
SIRT1endo-/-: endothelial knockout SIRT1
TGFβRIIendo+/-: TGFβ receptor II knockout
UUO: unilateral ureteral obstruction.
