# Supplementary material for: Fibrogenic Secretome of Sirtuin 1-Deficient Endothelial Cells: Wnt, Notch and Glycocalyx Rheostat
Source: Front Physiol. 2018 Sep 21;9:1325. doi: 10.3389/fphys.2018.01325 (PMC6160542; doi:10.3389/fphys.2018.01325)
Supplement: Supplementary file 1 [file Table_1.docx]

**List of identified protein in the endothelial secretome (proteins identified exclusively in RMVEC isolated from Sirt1^endo^-/- mice and treated with TGFß1)**

| **Accession** | **Protein Name** |
| --- | --- |
| PGK1_MOUSE | Phosphoglycerate kinase 1 OS=Mus musculus GN=Pgk1 PE=1 SV=4 |
| WDR1_MOUSE | WD repeat-containing protein 1 OS=Mus musculus GN=Wdr1 PE=1 SV=3 |
| IBP7_MOUSE | Insulin-like growth factor-binding protein 7 OS=Mus musculus GN=Igfbp7 PE=2 SV=3 |
| SPRC_MOUSE | SPARC OS=Mus musculus GN=Sparc PE=1 SV=1 |
| SODC_MOUSE | Superoxide dismutase [Cu-Zn] OS=Mus musculus GN=Sod1 PE=1 SV=2 |
| FRIL1_MOUSE | Ferritin light chain 1 OS=Mus musculus GN=Ftl1 PE=1 SV=2 |
| CALR_MOUSE | Calreticulin OS=Mus musculus GN=Calr PE=1 SV=1 |
| TKT_MOUSE | Transketolase OS=Mus musculus GN=Tkt PE=1 SV=1 |
| G6PI_MOUSE | Glucose-6-phosphate isomerase OS=Mus musculus GN=Gpi PE=1 SV=4 |
| LAMP2_MOUSE | Lysosome-associated membrane glycoprotein 2 OS=Mus musculus GN=Lamp2 PE=2 SV=2 |
| PDIA3_MOUSE | Protein disulfide-isomerase A3 OS=Mus musculus GN=Pdia3 PE=1 SV=2 |
| AATM_MOUSE | Aspartate aminotransferase, mitochondrial OS=Mus musculus GN=Got2 PE=1 SV=1 |
| PAI1_MOUSE | Plasminogen activator inhibitor 1 OS=Mus musculus GN=Serpine1 PE=1 SV=1 |
| MOES_MOUSE | Moesin OS=Mus musculus GN=Msn PE=1 SV=3 |
| PRDX6_MOUSE | Peroxiredoxin-6 OS=Mus musculus GN=Prdx6 PE=1 SV=3 |
| FSCN1_MOUSE | Fascin OS=Mus musculus GN=Fscn1 PE=1 SV=4 |
| CTGF_MOUSE | Connective tissue growth factor OS=Mus musculus GN=Ctgf PE=2 SV=3 |
| LEG1_MOUSE | Galectin-1 OS=Mus musculus GN=Lgals1 PE=1 SV=3 |
| TRXR1_MOUSE | Thioredoxin reductase 1, cytoplasmic OS=Mus musculus GN=Txnrd1 PE=1 SV=3 |
| CATL1_MOUSE | Cathepsin L1 OS=Mus musculus GN=Ctsl PE=1 SV=2 |
| AMPL_MOUSE | Cytosol aminopeptidase OS=Mus musculus GN=Lap3 PE=1 SV=3 |
| PTK7_MOUSE | Inactive tyrosine-protein kinase 7 OS=Mus musculus GN=Ptk7 PE=1 SV=1 |
| PDIA1_MOUSE | Protein disulfide-isomerase OS=Mus musculus GN=P4hb PE=1 SV=2 |
| CADH2_MOUSE | Cadherin-2 OS=Mus musculus GN=Cdh2 PE=1 SV=2 |
| PPIB_MOUSE | Peptidyl-prolyl cis-trans isomerase B OS=Mus musculus GN=Ppib PE=1 SV=2 |
| TIMP1_MOUSE | Metalloproteinase inhibitor 1 OS=Mus musculus GN=Timp1 PE=1 SV=2 |
| CALU_MOUSE | Calumenin OS=Mus musculus GN=Calu PE=1 SV=1 |
| KCRB_MOUSE | Creatine kinase B-type OS=Mus musculus GN=Ckb PE=1 SV=1 |
| TSP1_MOUSE | Thrombospondin-1 OS=Mus musculus GN=Thbs1 PE=1 SV=1 |
| CD44_MOUSE | CD44 antigen OS=Mus musculus GN=Cd44 PE=1 SV=3 |
| AK1A1_MOUSE | Alcohol dehydrogenase [NADP(+)] OS=Mus musculus GN=Akr1a1 PE=1 SV=3 |
| DHE3_MOUSE | Glutamate dehydrogenase 1, mitochondrial OS=Mus musculus GN=Glud1 PE=1 SV=1 |
| TPM4_MOUSE | Tropomyosin alpha-4 chain OS=Mus musculus GN=Tpm4 PE=2 SV=3 |
| AATC_MOUSE | Aspartate aminotransferase, cytoplasmic OS=Mus musculus GN=Got1 PE=1 SV=3 |
| EPCR_MOUSE | Endothelial protein C receptor OS=Mus musculus GN=Procr PE=2 SV=3 |
| PSA6_MOUSE | Proteasome subunit alpha type-6 OS=Mus musculus GN=Psma6 PE=1 SV=1 |
| GBLP_MOUSE | Guanine nucleotide-binding protein subunit beta-2-like 1 OS=Mus musculus GN=Gnb2l1 PE=1 SV=3 |
| BASP1_MOUSE | Brain acid soluble protein 1 OS=Mus musculus GN=Basp1 PE=1 SV=3 |
| PSA4_MOUSE | Proteasome subunit alpha type-4 OS=Mus musculus GN=Psma4 PE=1 SV=1 |
| FBLN2_MOUSE | Fibulin-2 OS=Mus musculus GN=Fbln2 PE=1 SV=2 |
| PSA7_MOUSE | Proteasome subunit alpha type-7 OS=Mus musculus GN=Psma7 PE=1 SV=1 |
| RSSA_MOUSE | 40S ribosomal protein SA OS=Mus musculus GN=Rpsa PE=1 SV=4 |
| CNDP2_MOUSE | Cytosolic non-specific dipeptidase OS=Mus musculus GN=Cndp2 PE=1 SV=1 |
| VCAM1_MOUSE | Vascular cell adhesion protein 1 OS=Mus musculus GN=Vcam1 PE=1 SV=1 |
| TENA_MOUSE | Tenascin OS=Mus musculus GN=Tnc PE=1 SV=1 |
| PSB1_MOUSE | Proteasome subunit beta type-1 OS=Mus musculus GN=Psmb1 PE=1 SV=1 |
| PSA2_MOUSE | Proteasome subunit alpha type-2 OS=Mus musculus GN=Psma2 PE=1 SV=3 |
| CADH5_MOUSE | Cadherin-5 OS=Mus musculus GN=Cdh5 PE=1 SV=2 |
| LG3BP_MOUSE | Galectin-3-binding protein OS=Mus musculus GN=Lgals3bp PE=1 SV=1 |
| TINAL_MOUSE | Tubulointerstitial nephritis antigen-like OS=Mus musculus GN=Tinagl1 PE=1 SV=1 |
| EPDR1_MOUSE | Mammalian ependymin-related protein 1 OS=Mus musculus GN=Epdr1 PE=2 SV=1 |
| PEPD_MOUSE | Xaa-Pro dipeptidase OS=Mus musculus GN=Pepd PE=2 SV=3 |
| BMP1_MOUSE | Bone morphogenetic protein 1 OS=Mus musculus GN=Bmp1 PE=1 SV=2 |
| ESTD_MOUSE | S-formylglutathione hydrolase OS=Mus musculus GN=Esd PE=1 SV=1 |
| CISY_MOUSE | Citrate synthase, mitochondrial OS=Mus musculus GN=Cs PE=1 SV=1 |
| ACON_MOUSE | Aconitate hydratase, mitochondrial OS=Mus musculus GN=Aco2 PE=1 SV=1 |
| DPP3_MOUSE | Dipeptidyl peptidase 3 OS=Mus musculus GN=Dpp3 PE=2 SV=2 |
| SEM7A_MOUSE | Semaphorin-7A OS=Mus musculus GN=Sema7a PE=1 SV=1 |
| MIF_MOUSE | Macrophage migration inhibitory factor OS=Mus musculus GN=Mif PE=1 SV=2 |
| PSB3_MOUSE | Proteasome subunit beta type-3 OS=Mus musculus GN=Psmb3 PE=1 SV=1 |
| JAG1_MOUSE | Protein jagged-1 OS=Mus musculus GN=Jag1 PE=1 SV=1 |
| ITA3_MOUSE | Integrin alpha-3 OS=Mus musculus GN=Itga3 PE=1 SV=1 |
| GPX3_MOUSE | Glutathione peroxidase 3 OS=Mus musculus GN=Gpx3 PE=2 SV=2 |
| FRIH_MOUSE | Ferritin heavy chain OS=Mus musculus GN=Fth1 PE=1 SV=2 |
| GALNS_MOUSE | N-acetylgalactosamine-6-sulfatase OS=Mus musculus GN=Galns PE=2 SV=2 |
| ADHX_MOUSE | Alcohol dehydrogenase class-3 OS=Mus musculus GN=Adh5 PE=1 SV=3 |
| LYAG_MOUSE | Lysosomal alpha-glucosidase OS=Mus musculus GN=Gaa PE=1 SV=2 |
| GSHR_MOUSE | Glutathione reductase, mitochondrial OS=Mus musculus GN=Gsr PE=1 SV=3 |
| ATPB_MOUSE | ATP synthase subunit beta, mitochondrial OS=Mus musculus GN=Atp5b PE=1 SV=2 |
| MUC18_MOUSE | Cell surface glycoprotein MUC18 OS=Mus musculus GN=Mcam PE=1 SV=1 |
| X3CL1_MOUSE | Fractalkine OS=Mus musculus GN=Cx3cl1 PE=2 SV=3 |
| MDHC_MOUSE | Malate dehydrogenase, cytoplasmic OS=Mus musculus GN=Mdh1 PE=1 SV=3 |
| ITB1_MOUSE | Integrin beta-1 OS=Mus musculus GN=Itgb1 PE=1 SV=1 |
| MYH9_MOUSE | Myosin-9 OS=Mus musculus GN=Myh9 PE=1 SV=4 |
| THIM_MOUSE | 3-ketoacyl-CoA thiolase, mitochondrial OS=Mus musculus GN=Acaa2 PE=1 SV=3 |
| CSF1_MOUSE | Macrophage colony-stimulating factor 1 OS=Mus musculus GN=Csf1 PE=1 SV=2 |
| MA2B1_MOUSE | Lysosomal alpha-mannosidase OS=Mus musculus GN=Man2b1 PE=2 SV=4 |
| RS3_MOUSE | 40S ribosomal protein S3 OS=Mus musculus GN=Rps3 PE=1 SV=1 |
| GSTO1_MOUSE | Glutathione S-transferase omega-1 OS=Mus musculus GN=Gsto1 PE=2 SV=2 |
| NID1_MOUSE | Nidogen-1 OS=Mus musculus GN=Nid1 PE=1 SV=2 |
| CO5A1_MOUSE | Collagen alpha-1(V) chain OS=Mus musculus GN=Col5a1 PE=2 SV=2 |
| LYVE1_MOUSE | Lymphatic vessel endothelial hyaluronic acid receptor 1 OS=Mus musculus GN=Lyve1 PE=1 SV=1 |
| RS21_MOUSE | 40S ribosomal protein S21 OS=Mus musculus GN=Rps21 PE=2 SV=1 |
| PSA_MOUSE | Puromycin-sensitive aminopeptidase OS=Mus musculus GN=Npepps PE=1 SV=2 |
| SODM_MOUSE | Superoxide dismutase [Mn], mitochondrial OS=Mus musculus GN=Sod2 PE=1 SV=3 |
| UFO_MOUSE | Tyrosine-protein kinase receptor UFO OS=Mus musculus GN=Axl PE=1 SV=2 |
| PNPH_MOUSE | Purine nucleoside phosphorylase OS=Mus musculus GN=Pnp PE=1 SV=2 |
| CADH6_MOUSE | Cadherin-6 OS=Mus musculus GN=Cdh6 PE=1 SV=2 |
| DDAH1_MOUSE | N(G),N(G)-dimethylarginine dimethylaminohydrolase 1 OS=Mus musculus GN=Ddah1 PE=1 SV=3 |
| PSB7_MOUSE | Proteasome subunit beta type-7 OS=Mus musculus GN=Psmb7 PE=1 SV=1 |
| RS3A_MOUSE | 40S ribosomal protein S3a OS=Mus musculus GN=Rps3a PE=1 SV=3 |
| RS28_MOUSE | 40S ribosomal protein S28 OS=Mus musculus GN=Rps28 PE=2 SV=1 |
| FLNC_MOUSE | Filamin-C OS=Mus musculus GN=Flnc PE=1 SV=3 |
| CTL2A_MOUSE | Protein CTLA-2-alpha OS=Mus musculus GN=Ctla2a PE=2 SV=2 |
| TCTP_MOUSE | Translationally-controlled tumor protein OS=Mus musculus GN=Tpt1 PE=1 SV=1 |
| NFH_MOUSE | Neurofilament heavy polypeptide OS=Mus musculus GN=Nefh PE=1 SV=3 |
| 1433F_MOUSE | 14-3-3 protein eta OS=Mus musculus GN=Ywhah PE=1 SV=2 |
| ADA_MOUSE | Adenosine deaminase OS=Mus musculus GN=Ada PE=1 SV=3 |
| SERC_MOUSE | Phosphoserine aminotransferase OS=Mus musculus GN=Psat1 PE=1 SV=1 |
| DKK3_MOUSE | Dickkopf-related protein 3 OS=Mus musculus GN=Dkk3 PE=2 SV=1 |
| GANAB_MOUSE | Neutral alpha-glucosidase AB OS=Mus musculus GN=Ganab PE=1 SV=1 |
| FRRS1_MOUSE | Ferric-chelate reductase 1 OS=Mus musculus GN=FRRS1 PE=1 SV=1 |
| ASAH1_MOUSE | Acid ceramidase OS=Mus musculus GN=Asah1 PE=1 SV=1 |
| A2M_MOUSE | Alpha-2-macroglobulin OS=Mus musculus GN=A2m PE=1 SV=3 |
| TBA1A_MOUSE | Tubulin alpha-1A chain OS=Mus musculus GN=Tuba1a PE=1 SV=1 |
| ACTN4_MOUSE | Alpha-actinin-4 OS=Mus musculus GN=Actn4 PE=1 SV=1 |
| RS20_MOUSE | 40S ribosomal protein S20 OS=Mus musculus GN=Rps20 PE=1 SV=1 |
| PPIC_MOUSE | Peptidyl-prolyl cis-trans isomerase C OS=Mus musculus GN=Ppic PE=1 SV=1 |
| RL30_MOUSE | 60S ribosomal protein L30 OS=Mus musculus GN=Rpl30 PE=2 SV=2 |
| ALDR_MOUSE | Aldose reductase OS=Mus musculus GN=Akr1b1 PE=1 SV=3 |
| COF1_MOUSE | Cofilin-1 OS=Mus musculus GN=Cfl1 PE=1 SV=3 |
| GDIA_MOUSE | Rab GDP dissociation inhibitor alpha OS=Mus musculus GN=Gdi1 PE=1 SV=3 |
| INHBA_MOUSE | Inhibin beta A chain OS=Mus musculus GN=Inhba PE=1 SV=1 |
| PPGB_MOUSE | Lysosomal protective protein OS=Mus musculus GN=Ctsa PE=1 SV=1 |
| MA2B2_MOUSE | Epididymis-specific alpha-mannosidase OS=Mus musculus GN=Man2b2 PE=2 SV=2 |
| SDCB1_MOUSE | Syntenin-1 OS=Mus musculus GN=Sdcbp PE=1 SV=1 |
| MARCS_MOUSE | Myristoylated alanine-rich C-kinase substrate OS=Mus musculus GN=Marcks PE=1 SV=2 |
| RS14_MOUSE | 40S ribosomal protein S14 OS=Mus musculus GN=Rps14 PE=2 SV=3 |
| FSTL1_MOUSE | Follistatin-related protein 1 OS=Mus musculus GN=Fstl1 PE=1 SV=2 |
| GSHB_MOUSE | Glutathione synthetase OS=Mus musculus GN=Gss PE=2 SV=1 |
| PUR9_MOUSE | Bifunctional purine biosynthesis protein PURH OS=Mus musculus GN=Atic PE=1 SV=2 |
| DOPD_MOUSE | D-dopachrome decarboxylase OS=Mus musculus GN=Ddt PE=1 SV=3 |
| ICAM1_MOUSE | Intercellular adhesion molecule 1 OS=Mus musculus GN=Icam1 PE=1 SV=1 |
| JAM1_MOUSE | Junctional adhesion molecule A OS=Mus musculus GN=F11r PE=1 SV=2 |
| NEST_MOUSE | Nestin OS=Mus musculus GN=Nes PE=1 SV=1 |
| ACTN1_MOUSE | Alpha-actinin-1 OS=Mus musculus GN=Actn1 PE=1 SV=1 |
| OAT_MOUSE | Ornithine aminotransferase, mitochondrial OS=Mus musculus GN=Oat PE=1 SV=1 |
| ANXA7_MOUSE | Annexin A7 OS=Mus musculus GN=Anxa7 PE=2 SV=2 |
| MYH10_MOUSE | Myosin-10 OS=Mus musculus GN=Myh10 PE=1 SV=2 |
| OSTP_MOUSE | Osteopontin OS=Mus musculus GN=Spp1 PE=1 SV=1 |
| BGLR_MOUSE | Beta-glucuronidase OS=Mus musculus GN=Gusb PE=2 SV=2 |
| PPCE_MOUSE | Prolyl endopeptidase OS=Mus musculus GN=Prep PE=2 SV=1 |
| CAD11_MOUSE | Cadherin-11 OS=Mus musculus GN=Cdh11 PE=1 SV=1 |
| 1433T_MOUSE | 14-3-3 protein theta OS=Mus musculus GN=Ywhaq PE=1 SV=1 |
| ALDH2_MOUSE | Aldehyde dehydrogenase, mitochondrial OS=Mus musculus GN=Aldh2 PE=1 SV=1 |
| CYTC_MOUSE | Cystatin-C OS=Mus musculus GN=Cst3 PE=2 SV=2 |
| FUMH_MOUSE | Fumarate hydratase, mitochondrial OS=Mus musculus GN=Fh PE=1 SV=3 |
| AKA12_MOUSE | A-kinase anchor protein 12 OS=Mus musculus GN=Akap12 PE=1 SV=1 |
| XPP1_MOUSE | Xaa-Pro aminopeptidase 1 OS=Mus musculus GN=Xpnpep1 PE=2 SV=1 |
| LDLR_MOUSE | Low-density lipoprotein receptor OS=Mus musculus GN=Ldlr PE=1 SV=2 |
| ACBP_MOUSE | Acyl-CoA-binding protein OS=Mus musculus GN=Dbi PE=1 SV=2 |
| RL11_MOUSE | 60S ribosomal protein L11 OS=Mus musculus GN=Rpl11 PE=1 SV=4 |
| NNRE_MOUSE | NAD(P)H-hydrate epimerase OS=Mus musculus GN=Apoa1bp PE=1 SV=1 |
| ARSA_MOUSE | Arylsulfatase A OS=Mus musculus GN=Arsa PE=2 SV=2 |
| LGUL_MOUSE | Lactoylglutathione lyase OS=Mus musculus GN=Glo1 PE=1 SV=3 |
| CK054_MOUSE | Ester hydrolase C11orf54 homolog OS=Mus musculus PE=2 SV=1 |
| LAMB1_MOUSE | Laminin subunit beta-1 OS=Mus musculus GN=Lamb1 PE=1 SV=3 |
| LRC15_MOUSE | Leucine-rich repeat-containing protein 15 OS=Mus musculus GN=Lrrc15 PE=2 SV=1 |
| VDAC2_MOUSE | Voltage-dependent anion-selective channel protein 2 OS=Mus musculus GN=Vdac2 PE=1 SV=2 |
| GDIB_MOUSE | Rab GDP dissociation inhibitor beta OS=Mus musculus GN=Gdi2 PE=1 SV=1 |
| TAGL_MOUSE | Transgelin OS=Mus musculus GN=Tagln PE=1 SV=3 |
| APEH_MOUSE | Acylamino-acid-releasing enzyme OS=Mus musculus GN=Apeh PE=2 SV=3 |
| FAAA_MOUSE | Fumarylacetoacetase OS=Mus musculus GN=Fah PE=1 SV=2 |
| B2MG_MOUSE | Beta-2-microglobulin OS=Mus musculus GN=B2m PE=1 SV=2 |
| FBLN1_MOUSE | Fibulin-1 OS=Mus musculus GN=Fbln1 PE=1 SV=2 |
| FLNB_MOUSE | Filamin-B OS=Mus musculus GN=Flnb PE=1 SV=3 |
| PTPRK_MOUSE | Receptor-type tyrosine-protein phosphatase kappa OS=Mus musculus GN=Ptprk PE=1 SV=1 |
| ACADL_MOUSE | Long-chain specific acyl-CoA dehydrogenase, mitochondrial OS=Mus musculus GN=Acadl PE=1 SV=2 |
| GPX1_MOUSE | Glutathione peroxidase 1 OS=Mus musculus GN=Gpx1 PE=1 SV=2 |
| RCN3_MOUSE | Reticulocalbin-3 OS=Mus musculus GN=Rcn3 PE=2 SV=1 |
| GBG12_MOUSE | Guanine nucleotide-binding protein G(I)/G(S)/G(O) subunit gamma-12 OS=Mus musculus GN=Gng12 PE=1 SV=3 |
| S10AD_MOUSE | Protein S100-A13 OS=Mus musculus GN=S100a13 PE=1 SV=1 |
| NIT2_MOUSE | Omega-amidase NIT2 OS=Mus musculus GN=Nit2 PE=1 SV=1 |
| C1QR1_MOUSE | Complement component C1q receptor OS=Mus musculus GN=Cd93 PE=1 SV=1 |
| CSTN1_MOUSE | Calsyntenin-1 OS=Mus musculus GN=Clstn1 PE=1 SV=1 |
| ITIH2_MOUSE | Inter-alpha-trypsin inhibitor heavy chain H2 OS=Mus musculus GN=Itih2 PE=1 SV=1 |
| PDXK_MOUSE | Pyridoxal kinase OS=Mus musculus GN=Pdxk PE=1 SV=1 |
| GUAD_MOUSE | Guanine deaminase OS=Mus musculus GN=Gda PE=1 SV=1 |
| HSP74_MOUSE | Heat shock 70 kDa protein 4 OS=Mus musculus GN=Hspa4 PE=1 SV=1 |
| NHLC3_MOUSE | NHL repeat-containing protein 3 OS=Mus musculus GN=Nhlrc3 PE=2 SV=1 |
| 6PGD_MOUSE | 6-phosphogluconate dehydrogenase, decarboxylating OS=Mus musculus GN=Pgd PE=1 SV=3 |
| ENPP5_MOUSE | Ectonucleotide pyrophosphatase/phosphodiesterase family member 5 OS=Mus musculus GN=Enpp5 PE=2 SV=3 |
| NUCB1_MOUSE | Nucleobindin-1 OS=Mus musculus GN=Nucb1 PE=1 SV=2 |
| HCDH_MOUSE | Hydroxyacyl-coenzyme A dehydrogenase, mitochondrial OS=Mus musculus GN=Hadh PE=1 SV=2 |
| ARSB_MOUSE | Arylsulfatase B OS=Mus musculus GN=Arsb PE=2 SV=3 |
| SET_MOUSE | Protein SET OS=Mus musculus GN=Set PE=1 SV=1 |
| LAMC1_MOUSE | Laminin subunit gamma-1 OS=Mus musculus GN=Lamc1 PE=1 SV=2 |
| MYL6_MOUSE | Myosin light polypeptide 6 OS=Mus musculus GN=Myl6 PE=1 SV=3 |
| CLUS_MOUSE | Clusterin OS=Mus musculus GN=Clu PE=1 SV=1 |
| PGAM1_MOUSE | Phosphoglycerate mutase 1 OS=Mus musculus GN=Pgam1 PE=1 SV=3 |
| RS4X_MOUSE | 40S ribosomal protein S4, X isoform OS=Mus musculus GN=Rps4x PE=2 SV=2 |
| GTPC1_MOUSE | Putative GTP cyclohydrolase 1 type 2 Nif3l1 OS=Mus musculus GN=Nif3l1 PE=1 SV=4 |
| MEMO1_MOUSE | Protein MEMO1 OS=Mus musculus GN=Memo1 PE=1 SV=1 |
| PCNA_MOUSE | Proliferating cell nuclear antigen OS=Mus musculus GN=Pcna PE=1 SV=2 |
| LDHB_MOUSE | L-lactate dehydrogenase B chain OS=Mus musculus GN=Ldhb PE=1 SV=2 |
| GLU2B_MOUSE | Glucosidase 2 subunit beta OS=Mus musculus GN=Prkcsh PE=1 SV=1 |
| CH10_MOUSE | 10 kDa heat shock protein, mitochondrial OS=Mus musculus GN=Hspe1 PE=1 SV=2 |
| PSB8_MOUSE | Proteasome subunit beta type-8 OS=Mus musculus GN=Psmb8 PE=1 SV=2 |
| MFGM_MOUSE | Lactadherin OS=Mus musculus GN=Mfge8 PE=1 SV=3 |
| FCL_MOUSE | GDP-L-fucose synthase OS=Mus musculus GN=Tsta3 PE=2 SV=3 |
| GRN_MOUSE | Granulins OS=Mus musculus GN=Grn PE=1 SV=2 |
| HS90B_MOUSE | Heat shock protein HSP 90-beta OS=Mus musculus GN=Hsp90ab1 PE=1 SV=3 |
| SAHH_MOUSE | Adenosylhomocysteinase OS=Mus musculus GN=Ahcy PE=1 SV=3 |
| HBEGF_MOUSE | Proheparin-binding EGF-like growth factor OS=Mus musculus GN=Hbegf PE=2 SV=1 |
| CADM1_MOUSE | Cell adhesion molecule 1 OS=Mus musculus GN=Cadm1 PE=1 SV=2 |
| RAB7A_MOUSE | Ras-related protein Rab-7a OS=Mus musculus GN=Rab7a PE=1 SV=2 |
| S10AA_MOUSE | Protein S100-A10 OS=Mus musculus GN=S100a10 PE=1 SV=2 |
| VDAC1_MOUSE | Voltage-dependent anion-selective channel protein 1 OS=Mus musculus GN=Vdac1 PE=1 SV=3 |
| ITA5_MOUSE | Integrin alpha-5 OS=Mus musculus GN=Itga5 PE=1 SV=3 |
| CAH2_MOUSE | Carbonic anhydrase 2 OS=Mus musculus GN=Ca2 PE=1 SV=4 |
| NCAM1_MOUSE | Neural cell adhesion molecule 1 OS=Mus musculus GN=Ncam1 PE=1 SV=3 |
| CO4A2_MOUSE | Collagen alpha-2(IV) chain OS=Mus musculus GN=Col4a2 PE=2 SV=4 |
| DDB1_MOUSE | DNA damage-binding protein 1 OS=Mus musculus GN=Ddb1 PE=1 SV=2 |
| MVP_MOUSE | Major vault protein OS=Mus musculus GN=Mvp PE=1 SV=4 |
| ESM1_MOUSE | Endothelial cell-specific molecule 1 OS=Mus musculus GN=Esm1 PE=2 SV=1 |
| DHPR_MOUSE | Dihydropteridine reductase OS=Mus musculus GN=Qdpr PE=1 SV=2 |
| SEM5A_MOUSE | Semaphorin-5A OS=Mus musculus GN=Sema5a PE=2 SV=1 |
| ACADM_MOUSE | Medium-chain specific acyl-CoA dehydrogenase, mitochondrial OS=Mus musculus GN=Acadm PE=1 SV=1 |
| GPNMB_MOUSE | Transmembrane glycoprotein NMB OS=Mus musculus GN=Gpnmb PE=1 SV=2 |
| AMPE_MOUSE | Glutamyl aminopeptidase OS=Mus musculus GN=Enpep PE=1 SV=1 |
| MANBA_MOUSE | Beta-mannosidase OS=Mus musculus GN=Manba PE=2 SV=1 |
| ASAH2_MOUSE | Neutral ceramidase OS=Mus musculus GN=Asah2 PE=1 SV=1 |
| ATRN_MOUSE | Attractin OS=Mus musculus GN=Atrn PE=2 SV=3 |
| PPAL_MOUSE | Lysosomal acid phosphatase OS=Mus musculus GN=Acp2 PE=2 SV=2 |
| NRCAM_MOUSE | Neuronal cell adhesion molecule OS=Mus musculus GN=Nrcam PE=1 SV=2 |
| SSRD_MOUSE | Translocon-associated protein subunit delta OS=Mus musculus GN=Ssr4 PE=2 SV=1 |
| LOXL2_MOUSE | Lysyl oxidase homolog 2 OS=Mus musculus GN=Loxl2 PE=1 SV=2 |
| CO4A1_MOUSE | Collagen alpha-1(IV) chain OS=Mus musculus GN=Col4a1 PE=2 SV=4 |
| RL7A_MOUSE | 60S ribosomal protein L7a OS=Mus musculus GN=Rpl7a PE=1 SV=2 |
| HEXA_MOUSE | Beta-hexosaminidase subunit alpha OS=Mus musculus GN=Hexa PE=2 SV=2 |
| AGAL_MOUSE | Alpha-galactosidase A OS=Mus musculus GN=Gla PE=1 SV=1 |
| RS17_MOUSE | 40S ribosomal protein S17 OS=Mus musculus GN=Rps17 PE=1 SV=2 |
| DNPEP_MOUSE | Aspartyl aminopeptidase OS=Mus musculus GN=Dnpep PE=2 SV=2 |
| P3IP1_MOUSE | Phosphoinositide-3-kinase-interacting protein 1 OS=Mus musculus GN=Pik3ip1 PE=2 SV=1 |
| JAG2_MOUSE | Protein jagged-2 OS=Mus musculus GN=Jag2 PE=2 SV=2 |
| CLIC1_MOUSE | Chloride intracellular channel protein 1 OS=Mus musculus GN=Clic1 PE=1 SV=3 |
| CAZA1_MOUSE | F-actin-capping protein subunit alpha-1 OS=Mus musculus GN=Capza1 PE=1 SV=4 |
| APOB_MOUSE | Apolipoprotein B-100 OS=Mus musculus GN=Apob PE=1 SV=1 |
| GALM_MOUSE | Aldose 1-epimerase OS=Mus musculus GN=Galm PE=2 SV=1 |
| SCOT1_MOUSE | Succinyl-CoA:3-ketoacid coenzyme A transferase 1, mitochondrial OS=Mus musculus GN=Oxct1 PE=1 SV=1 |
| ES1_MOUSE | ES1 protein homolog, mitochondrial OS=Mus musculus GN=D10Jhu81e PE=1 SV=1 |
| RLA2_MOUSE | 60S acidic ribosomal protein P2 OS=Mus musculus GN=Rplp2 PE=1 SV=3 |
| ENPL_MOUSE | Endoplasmin OS=Mus musculus GN=Hsp90b1 PE=1 SV=2 |
| RS7_MOUSE | 40S ribosomal protein S7 OS=Mus musculus GN=Rps7 PE=2 SV=1 |
| ENPP4_MOUSE | Bis(5'-adenosyl)-triphosphatase enpp4 OS=Mus musculus GN=Enpp4 PE=1 SV=1 |
| SHPS1_MOUSE | Tyrosine-protein phosphatase non-receptor type substrate 1 OS=Mus musculus GN=Sirpa PE=1 SV=1 |
| AP4A_MOUSE | Bis(5'-nucleosyl)-tetraphosphatase [asymmetrical] OS=Mus musculus GN=Nudt2 PE=1 SV=3 |
| DEST_MOUSE | Destrin OS=Mus musculus GN=Dstn PE=1 SV=3 |
| CATC_MOUSE | Dipeptidyl peptidase 1 OS=Mus musculus GN=Ctsc PE=2 SV=1 |
| NICA_MOUSE | Nicastrin OS=Mus musculus GN=Ncstn PE=1 SV=3 |
| TXND5_MOUSE | Thioredoxin domain-containing protein 5 OS=Mus musculus GN=Txndc5 PE=1 SV=2 |
| DDAH2_MOUSE | N(G),N(G)-dimethylarginine dimethylaminohydrolase 2 OS=Mus musculus GN=Ddah2 PE=1 SV=1 |
| PGP_MOUSE | Phosphoglycolate phosphatase OS=Mus musculus GN=Pgp PE=1 SV=1 |
| MEG10_MOUSE | Multiple epidermal growth factor-like domains protein 10 OS=Mus musculus GN=Megf10 PE=1 SV=1 |
| GRAE_MOUSE | Granzyme E OS=Mus musculus GN=Gzme PE=1 SV=1 |
| RADI_MOUSE | Radixin OS=Mus musculus GN=Rdx PE=1 SV=3 |
| DIAC_MOUSE | Di-N-acetylchitobiase OS=Mus musculus GN=Ctbs PE=2 SV=2 |
| HEXB_MOUSE | Beta-hexosaminidase subunit beta OS=Mus musculus GN=Hexb PE=2 SV=2 |
| THOP1_MOUSE | Thimet oligopeptidase OS=Mus musculus GN=Thop1 PE=1 SV=1 |
| MRP_MOUSE | MARCKS-related protein OS=Mus musculus GN=Marcksl1 PE=1 SV=2 |
| ITAV_MOUSE | Integrin alpha-V OS=Mus musculus GN=Itgav PE=1 SV=2 |
| GBB2_MOUSE | Guanine nucleotide-binding protein G(I)/G(S)/G(T) subunit beta-2 OS=Mus musculus GN=Gnb2 PE=1 SV=3 |
| MTPN_MOUSE | Myotrophin OS=Mus musculus GN=Mtpn PE=1 SV=2 |
| ACADS_MOUSE | Short-chain specific acyl-CoA dehydrogenase, mitochondrial OS=Mus musculus GN=Acads PE=1 SV=2 |
| A2MP_MOUSE | Alpha-2-macroglobulin-P OS=Mus musculus GN=A2mp PE=2 SV=2 |
| BGAL_MOUSE | Beta-galactosidase OS=Mus musculus GN=Glb1 PE=2 SV=1 |
| CYR61_MOUSE | Protein CYR61 OS=Mus musculus GN=Cyr61 PE=1 SV=1 |
| FKB1A_MOUSE | Peptidyl-prolyl cis-trans isomerase FKBP1A OS=Mus musculus GN=Fkbp1a PE=1 SV=2 |
| BAF_MOUSE | Barrier-to-autointegration factor OS=Mus musculus GN=Banf1 PE=1 SV=1 |
| CYTB_MOUSE | Cystatin-B OS=Mus musculus GN=Cstb PE=1 SV=1 |
| BCAT1_MOUSE | Branched-chain-amino-acid aminotransferase, cytosolic OS=Mus musculus GN=Bcat1 PE=2 SV=2 |
| RL38_MOUSE | 60S ribosomal protein L38 OS=Mus musculus GN=Rpl38 PE=2 SV=3 |
| RLA0_MOUSE | 60S acidic ribosomal protein P0 OS=Mus musculus GN=Rplp0 PE=1 SV=3 |
| CLCA_MOUSE | Clathrin light chain A OS=Mus musculus GN=Clta PE=1 SV=2 |
| SC31B_MOUSE | Protein transport protein Sec31B OS=Mus musculus GN=Sec31b PE=2 SV=2 |
| HEP2_MOUSE | Heparin cofactor 2 OS=Mus musculus GN=Serpind1 PE=1 SV=1 |
| ATOX1_MOUSE | Copper transport protein ATOX1 OS=Mus musculus GN=Atox1 PE=1 SV=1 |
| LASP1_MOUSE | LIM and SH3 domain protein 1 OS=Mus musculus GN=Lasp1 PE=1 SV=1 |
| PTPRM_MOUSE | Receptor-type tyrosine-protein phosphatase mu OS=Mus musculus GN=Ptprm PE=2 SV=2 |
| MUG1_MOUSE | Murinoglobulin-1 OS=Mus musculus GN=Mug1 PE=1 SV=3 |
| TLL1_MOUSE | Tolloid-like protein 1 OS=Mus musculus GN=Tll1 PE=1 SV=1 |
| ALDOC_MOUSE | Fructose-bisphosphate aldolase C OS=Mus musculus GN=Aldoc PE=1 SV=4 |
| HS90A_MOUSE | Heat shock protein HSP 90-alpha OS=Mus musculus GN=Hsp90aa1 PE=1 SV=4 |
| GBB1_MOUSE | Guanine nucleotide-binding protein G(I)/G(S)/G(T) subunit beta-1 OS=Mus musculus GN=Gnb1 PE=1 SV=3 |
| GAPR1_MOUSE | Golgi-associated plant pathogenesis-related protein 1 OS=Mus musculus GN=Glipr2 PE=2 SV=3 |
| PTPS_MOUSE | 6-pyruvoyl tetrahydrobiopterin synthase OS=Mus musculus GN=Pts PE=2 SV=2 |
| PA1B2_MOUSE | Platelet-activating factor acetylhydrolase IB subunit beta OS=Mus musculus GN=Pafah1b2 PE=1 SV=2 |
| CAPZB_MOUSE | F-actin-capping protein subunit beta OS=Mus musculus GN=Capzb PE=1 SV=3 |
| 6PGL_MOUSE | 6-phosphogluconolactonase OS=Mus musculus GN=Pgls PE=2 SV=1 |
| DESM_MOUSE | Desmin OS=Mus musculus GN=Des PE=1 SV=3 |
| RS27L_MOUSE | 40S ribosomal protein S27-like OS=Mus musculus GN=Rps27l PE=2 SV=3 |
| PHP14_MOUSE | 14 kDa phosphohistidine phosphatase OS=Mus musculus GN=Phpt1 PE=2 SV=1 |
| PDC6I_MOUSE | Programmed cell death 6-interacting protein OS=Mus musculus GN=Pdcd6ip PE=1 SV=3 |
| PHS_MOUSE | Pterin-4-alpha-carbinolamine dehydratase OS=Mus musculus GN=Pcbd1 PE=1 SV=2 |
| NTF2_MOUSE | Nuclear transport factor 2 OS=Mus musculus GN=Nutf2 PE=2 SV=1 |
| PTPRG_MOUSE | Receptor-type tyrosine-protein phosphatase gamma OS=Mus musculus GN=Ptprg PE=1 SV=1 |
| CAPG_MOUSE | Macrophage-capping protein OS=Mus musculus GN=Capg PE=1 SV=2 |
| RS12_MOUSE | 40S ribosomal protein S12 OS=Mus musculus GN=Rps12 PE=1 SV=2 |
| CLH1_MOUSE | Clathrin heavy chain 1 OS=Mus musculus GN=Cltc PE=1 SV=3 |
| ARPC4_MOUSE | Actin-related protein 2/3 complex subunit 4 OS=Mus musculus GN=Arpc4 PE=1 SV=3 |
| LCAP_MOUSE | Leucyl-cystinyl aminopeptidase OS=Mus musculus GN=Lnpep PE=1 SV=1 |
| SDC4_MOUSE | Syndecan-4 OS=Mus musculus GN=Sdc4 PE=1 SV=1 |
| CD166_MOUSE | CD166 antigen OS=Mus musculus GN=Alcam PE=1 SV=3 |
| GFAP_MOUSE | Glial fibrillary acidic protein OS=Mus musculus GN=Gfap PE=1 SV=4 |
| CATH_MOUSE | Pro-cathepsin H OS=Mus musculus GN=Ctsh PE=2 SV=2 |
| DNAS1_MOUSE | Deoxyribonuclease-1 OS=Mus musculus GN=Dnase1 PE=2 SV=2 |
| CGL_MOUSE | Cystathionine gamma-lyase OS=Mus musculus GN=Cth PE=1 SV=1 |
| PECA1_MOUSE | Platelet endothelial cell adhesion molecule OS=Mus musculus GN=Pecam1 PE=1 SV=1 |
| FST_MOUSE | Follistatin OS=Mus musculus GN=Fst PE=2 SV=1 |
| HDHD2_MOUSE | Haloacid dehalogenase-like hydrolase domain-containing protein 2 OS=Mus musculus GN=Hdhd2 PE=1 SV=2 |
| APLP2_MOUSE | Amyloid-like protein 2 OS=Mus musculus GN=Aplp2 PE=1 SV=4 |
| ODPA_MOUSE | Pyruvate dehydrogenase E1 component subunit alpha, somatic form, mitochondrial OS=Mus musculus GN=Pdha1 PE=1 SV=1 |
| ECI1_MOUSE | Enoyl-CoA delta isomerase 1, mitochondrial OS=Mus musculus GN=Eci1 PE=1 SV=2 |
| RL24_MOUSE | 60S ribosomal protein L24 OS=Mus musculus GN=Rpl24 PE=1 SV=2 |
| NRP1_MOUSE | Neuropilin-1 OS=Mus musculus GN=Nrp1 PE=1 SV=2 |
| RL3_MOUSE | 60S ribosomal protein L3 OS=Mus musculus GN=Rpl3 PE=1 SV=3 |
| PGAM2_MOUSE | Phosphoglycerate mutase 2 OS=Mus musculus GN=Pgam2 PE=1 SV=3 |
| TSP2_MOUSE | Thrombospondin-2 OS=Mus musculus GN=Thbs2 PE=1 SV=2 |
| RINI_MOUSE | Ribonuclease inhibitor OS=Mus musculus GN=Rnh1 PE=1 SV=1 |
| ACTN2_MOUSE | Alpha-actinin-2 OS=Mus musculus GN=Actn2 PE=1 SV=2 |
| CRIM1_MOUSE | Cysteine-rich motor neuron 1 protein OS=Mus musculus GN=Crim1 PE=2 SV=2 |
| HVM53_MOUSE | Ig heavy chain V region RF OS=Mus musculus PE=1 SV=1 |
| IDH3A_MOUSE | Isocitrate dehydrogenase [NAD] subunit alpha, mitochondrial OS=Mus musculus GN=Idh3a PE=1 SV=1 |
| AMPB_MOUSE | Aminopeptidase B OS=Mus musculus GN=Rnpep PE=2 SV=2 |
| BLVRB_MOUSE | Flavin reductase (NADPH) OS=Mus musculus GN=Blvrb PE=2 SV=3 |
| PLMN_MOUSE | Plasminogen OS=Mus musculus GN=Plg PE=1 SV=3 |
| ACTN3_MOUSE | Alpha-actinin-3 OS=Mus musculus GN=Actn3 PE=2 SV=1 |
| PPIL1_MOUSE | Peptidyl-prolyl cis-trans isomerase-like 1 OS=Mus musculus GN=Ppil1 PE=2 SV=1 |
| MMRN2_MOUSE | Multimerin-2 OS=Mus musculus GN=Mmrn2 PE=2 SV=1 |
| PERI_MOUSE | Peripherin OS=Mus musculus GN=Prph PE=1 SV=2 |
| GLO2_MOUSE | Hydroxyacylglutathione hydrolase, mitochondrial OS=Mus musculus GN=Hagh PE=1 SV=2 |
| RS27_MOUSE | 40S ribosomal protein S27 OS=Mus musculus GN=Rps27 PE=1 SV=3 |
| RAB5C_MOUSE | Ras-related protein Rab-5C OS=Mus musculus GN=Rab5c PE=1 SV=2 |
| RL37A_MOUSE | 60S ribosomal protein L37a OS=Mus musculus GN=Rpl37a PE=2 SV=2 |
| CRLF1_MOUSE | Cytokine receptor-like factor 1 OS=Mus musculus GN=Crlf1 PE=1 SV=1 |
| INO1_MOUSE | Inositol-3-phosphate synthase 1 OS=Mus musculus GN=Isyna1 PE=2 SV=1 |
| RBM3_MOUSE | RNA-binding protein 3 OS=Mus musculus GN=Rbm3 PE=2 SV=1 |
| VAS1_MOUSE | V-type proton ATPase subunit S1 OS=Mus musculus GN=Atp6ap1 PE=1 SV=1 |
| K1C39_MOUSE | Keratin, type I cytoskeletal 39 OS=Mus musculus GN=Krt39 PE=2 SV=2 |
| TAGL3_MOUSE | Transgelin-3 OS=Mus musculus GN=Tagln3 PE=1 SV=1 |

OS=OrganismName; GN=GeneName; PE=ProteinExistence SV=SequenceVersion
